# The potential effect of temporary immunity as a result of bias associated with healthy users and social determinants on observations of influenza vaccine effectiveness; could unmeasured confounding explain observed links between seasonal influenza vaccine and pandemic H1N1 infection?

**DOI:** 10.1186/1471-2458-12-458

**Published:** 2012-06-20

**Authors:** Natasha S Crowcroft, Laura C Rosella

**Affiliations:** 1 Public Health Ontario, Toronto, Ontario, Canada, 480 University Avenue, Suite 300, Toronto, M5G 1 V2, ON, Canada; 2 Dalla Lana School of Public Health, University of Toronto, Toronto, ON, Canada; 3 Laboratory Medicine and Pathobiology, University of Toronto, Toronto, ON, Canada

**Keywords:** Influenza, Influenza vaccination, Confounding, Epidemiology

## Abstract

**Background:**

Five observational studies from Canada found an association between seasonal influenza vaccine receipt and increased risk of pandemic influenza H1N1 2009 infection. This association remains unexplained. Although uncontrolled confounding has been suggested as a possible explanation, the nature of such confounding has not been identified. Observational studies of influenza vaccination can be affected by confounding due to healthy users and the influence of social determinants on health. The purpose of this study was to investigate the influence that these two potential confounders may have in combination with temporary immunity, using stratified tables. The hypothesis is that respiratory virus infections may activate a temporary immunity that provides short-term non-specific protection against influenza and that the relationship with being a healthy user or having a social determinant may result in confounding.

**Methods:**

We simulated the effect of confounding on vaccine effectiveness assuming that this could result from both social determinants and healthy user effects as they both influence the risk of seasonal influenza and non-influenza respiratory virus infections as well as the likelihood of being vaccinated. We then examined what impact this may have had on measurement of seasonal influenza vaccine effectiveness against pandemic influenza.

**Results:**

In this simulation, failure to adjust for healthy users and social determinants would result in an erroneously increased risk of pandemic influenza infection associated with seasonal influenza vaccination. The effect sizes were not however large.

**Conclusions:**

We found that unmeasured healthy user effects and social determinants could result in an apparent association between seasonal influenza vaccine and pandemic influenza infection by virtue of being related to temporary immunity. Adjustment for social determinants of health and the healthy user effects are required in order to improve the quality of observational studies of influenza vaccine effectiveness.

## Background

Five observational studies carried out during the 2009 pandemic of influenza A H1N1 (pH1N1) in Canada found that previous 2008-9 seasonal influenza vaccine appeared to increase risk of being infected with the pandemic virus [1,2]. No significant methodological flaws were identified; unmeasured confounding was, however, the most common explanation for the observed association. Observational studies of seasonal influenza vaccination have been shown to be affected by confounding due to healthy-user effects and social determinants [3,4]. Such unmeasured confounding is unlikely to be the whole explanation given the prevalence and strength of association needed to explain the observations [5]. Heath Kelly and colleagues demonstrated a potential biological mechanism to explain the findings [6] in an elegant analysis. They postulated that a temporary cross-protective effect against pandemic influenza might have resulted from increased rates of seasonal influenza infection occurring in unvaccinated individuals in the months just prior to the start of the pandemic, when seasonal influenza was indeed circulating in Canada. They acknowledged however that the resulting effect sizes using realistic parameters were not large enough to explain what was observed in Canada. The idea of a temporary cross-protective effect is however consistent with no similar risk being reported by the same Canadian partnership in the second pandemic wave in Canada [7].

Cross-protection between influenza strains may have a number of mechanisms that could result in such a finding. Before carrying out expensive and invasive experiments to look for such biological effects it can be helpful to explore in simulations non-biological explanations. We carried out an exploration of how activation of a temporary cross-reactive immune response might explain the findings if it were an effect of Influenza-like-illness (ILI) caused by any respiratory infection (not solely influenza) operating via confounding by healthy-user effects and social determinants. Healthy users are more likely to be vaccinated and also less likely to acquire seasonal influenza making them potentially more at risk for pH1N1 due to the fact that they would be less likely to have temporary immunity. On the other spectrum, social deprivation has been shown to be related to a reduced uptake to seasonal influenza vaccination [8,9] as well as an increased risk of becoming ill due to respiratory infections including influenza [Lowcock EC et al. in press] [10]. We aimed to recreate the observed link between seasonal vaccine and pandemic H1N1 in a case control study assuming that the underlying distribution of individuals in social determinants and healthy user categories would have differential effects on both vaccine uptake and as a result in protective temporary immunity that could explain the observed positive association.

## Methods

**Figure 1:**
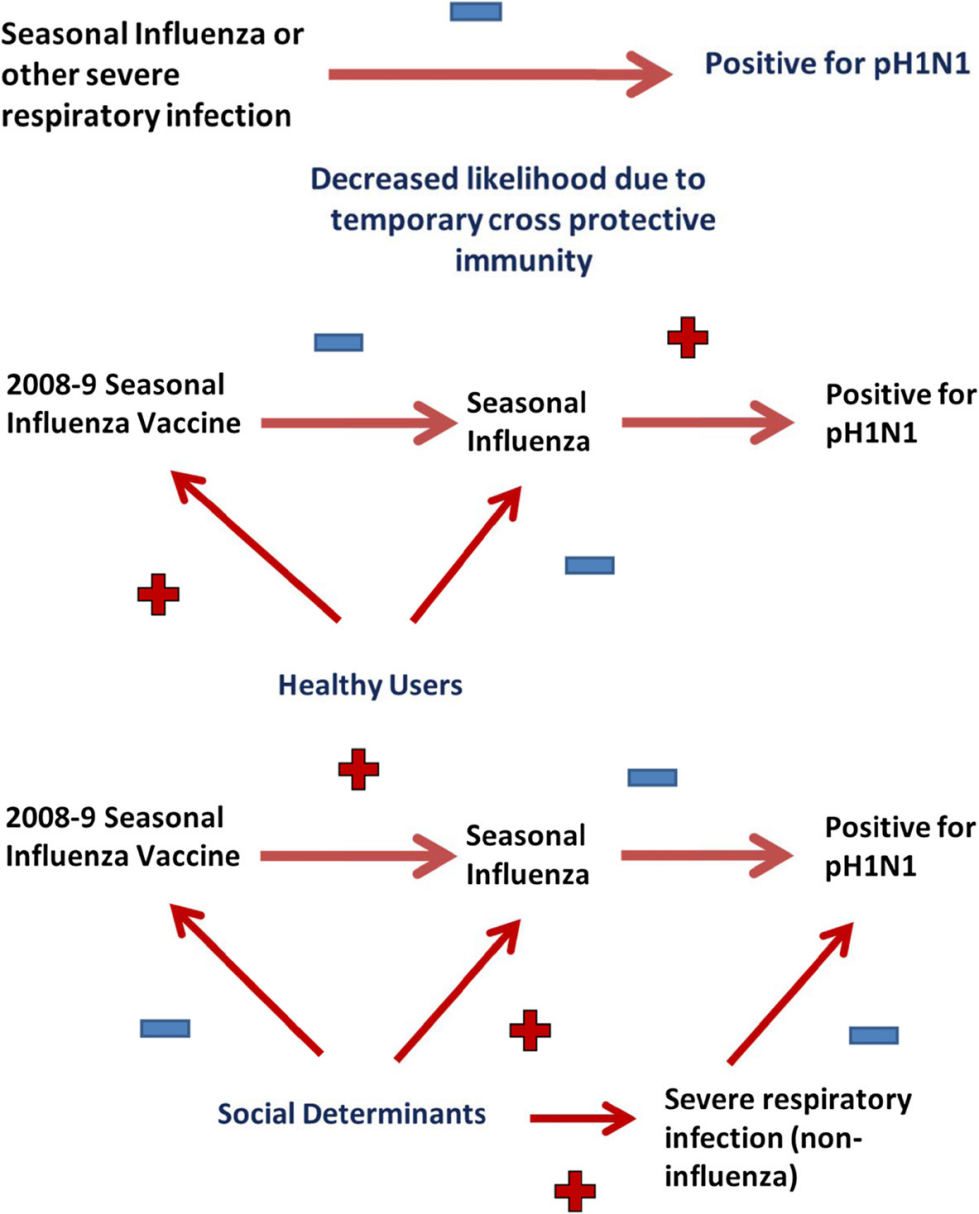
Effect of temporary immunity and biases associated with seasonal influenza vaccination.

**Figure 2:**
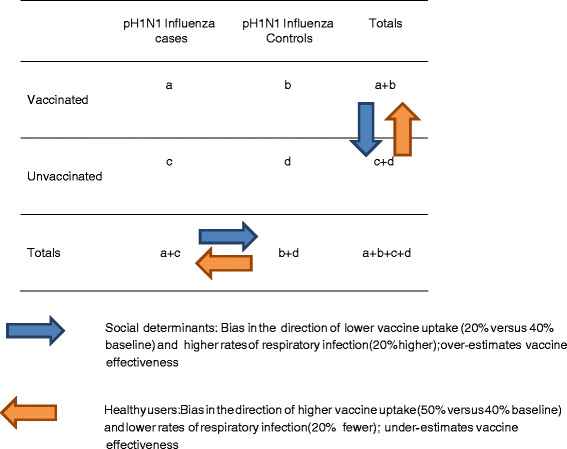
A standard two-by-two table with a demonstration of the different effects of the healthy user effect and social determinants on the distribution of cases and controls and their vaccination status.

We defined a confounder as a third factor which is related to both exposure (seasonal influenza vaccination) and outcome (pandemic influenza), and accounts for some or all of the observed relationship between the two (Figure 1). We constructed stratified results of a hypothetical case control study of 1000 subjects with a study design ratio of cases to controls of 1:1 (i.e. 500 in each group). In order to investigate the role of two unmeasured confounders, we stratified the case control population by healthy users (yes or no) and social determinants (present or not). To construct the underlying pre-pandemic distribution we assumed the effect of social determinants is to make people both less likely to be vaccinated and more likely to get sick and tested for seasonal influenza and other respiratory infections (confounding that results in a bias in favour of the seasonal vaccine). In a standard 2 by 2 table (Figure 2) comparing seasonal influenza vaccination status and pH1N1 case/control status, this would move people from cell a to c because we would expect to have recruited more unvaccinated cases with social determinants. We used an effect size that would drop vaccination coverage among those with the social determinants from 40% to 20%, potentially doubling risk of influenza (Table 1) and assumed this social determinants effect to be present in 20% of the population (Lowcock EC *et al.* In Press). We assumed that healthy users are more likely to be vaccinated and less likely to get seasonal influenza (Figure 2). This confounding also results in a bias in favour of vaccine, and moves people from cell d to b in the 2 by 2 because we would expect to have recruited more healthy users. We used an effect size of an absolute increase by 10% in seasonal influenza vaccine coverage among healthy users and a prevalence of 32% in the population, estimated on the basis of this being around half of the population that remains after excluding the 30% reporting a chronic disease [11]. For simplicity, we assumed that healthy users and those with social determinants are mutually exclusive and that case and control distribution remains 1:1 overall. We assumed that non-influenza respiratory infection would have the same qualitative effect on temporary immunity as influenza, whereby higher rates of both influenza and non-influenza respiratory infection caused by social determinants would increase protection from subsequent pH1N1 and lower rates of seasonal and non-influenza respiratory infections would increase susceptibility of healthy users. We set the impact of both influenza and non-influenza respiratory infections on temporary immunity equivalent to a 10% change in the distribution of cases and controls within a stratum. Baseline vaccination coverage was taken to be 40% based on Ontario’s Universal Influenza Immunization Program (UIIP) [12]. We assumed that being a healthy user does not however directly reduce the risk of pH1N1 because this is a susceptible population and so the force of infection would be high in most age groups. We ignored the impact of pre-existing immunity in older age groups [13]. 

**Table 1:** Baseline parameter assumptions

**Characteristic**	**Parameter**	
Proportions in population	Proportion of the population with a social determinant that would confound the association)	12%
	Proportion of the population classified as healthy users	40%
	Seasonal vaccine baseline coverage	40%
Effect sizes	Vaccination coverage among the population with social determinants (half baseline coverage)	10%
	Proportion of controls that would be classified as cases due to having social determinants	20%
	Vaccination coverage among population of healthy users (10% above baseline)	50%
	Proportion of cases that would be classified as controls due to being a healthy users	20%
	Seasonal influenza vaccine effectiveness against seasonal influenza	60%
	Additional effect of non-influenza ILI	10%

The baseline assumptions used to create the pre-pandemic population are shown in Table 1. We set the seasonal vaccine vaccine effectiveness at approximately 60% and assumed it had no effect on the risk of acquiring pandemic H1N1 (i.e. odds ratio equal to 1). The odds ratio derived from the summary Table 2 is deliberately calculated incorrectly, such that the effect of confounding by healthy users and social determinants is ignored, and thereby demonstrates what would be observed if confounding were not taken into account. We then calculated the odds ratio correctly using the Mantel Haenszel method to show the impact of accounting for these confounding factors [14]. 

**Table 2:** Seasonal influenza vaccination and H1N1 infection stratified by healthy users and social determinants

						
Healthy User = 0	Social Determinant = 1		H1N1		Total	
			+	-		
	Vaccinated	+	12	28	40	
		-	48	112	160	
			60	140	200	
Healthy User = 1	Social Determinant = 1		H1N1		Total	
			+	-		
	Vaccinated	+	140	60	200	
		-	140	60	200	
			280	120	400	
Healthy User = 0	Social Determinant = 0		H1N1		Total	
			+	-		
	Vaccinated	+	64	96	160	
		-	96	144	240	
			160	240	400	
**Summary Table**						
			H1N1		Total	
			+	-		
	Vaccinated	+	216	184	400	OR^§^ =1.3
		-	284	316	600	95% CI: 1.0 to 1.7
			500	500	1000	OR _M-H_ = 1.0
						95% CI: 0.8 to 1.3

We stratified the 500 cases and 500 controls based on the assumptions in Table 1 and re-constructed the vaccine effectiveness that was observed previously for seasonal influenza [3]. We populated the 2 by 2 tables between seasonal influenza vaccine and pandemic H1N1 influenza based on the hypothetical scenario that the unmeasured effects of healthy users and social determinants would influence both vaccine uptake and likelihood of acquiring temporary immunity through seasonal influenza infection and non-influenza respiratory infections prior to the pandemic.

## Results

Using the baseline assumptions to re-create the post-pH1N1 wave 1 associations and assumptions about the prevalence of healthy users and those with social determinants, an unadjusted odds ratio of 1.3 (95% CI: 1.0 to 1.7) would result between seasonal influenza vaccine and pandemic infection. The two confounding factors each had their own independent influence on pH1N1 based on our assumptions such that healthy users were twice as likely to be vaccinated against seasonal influenza (OR = 2.0, 95% CI: 1.5 to 2.6) and four times more likely to test positive for pH1N1 (OR = 4.0, 95% CI: 3.1 - 5.3). Those with social determinants were 70% less likely to be vaccinated against seasonal influenza (OR = 0.31, 95% CI: 0.21 to 0.44) and 65% less likely to test positive for pH1N1 (OR = 0.35, 95% CI: (0.25 TO 0.49) due to cross-protected immunity. Stratum-specific odds ratios are equal to 1 due to the fact that we assumed no effect modification. After accounting for the unmeasured effects of being a healthy user and having social determinants using the Mantel Haenszel method, this association would be reduced from 1.3 to 1.0 with the final association being non-significant (95% CI: 0.8 to 1.3). Increasing the sample size to 10,000 did slightly reduce the confidence intervals (Unadjusted OR = 1.3 95% CI: 1.2 to 1.4) but did not change the overall finding. We ran a secondary analysis assuming various levels of co-occurrence between social determinants and healthy users but because the effects were opposing each other this did not significantly change the overall finding. Furthermore, these confounders are unlikely to co-occur so any influence would be expected to be minimal.

## Discussion

The potential for unmeasured confounding exists in any observational design. Exploring the nature of this uncertainty is important to the interpretation of observational studies to assess vaccine effectiveness [15]. This study demonstrates a simple way to reveal the potential impact of unmeasured confounding that is related to temporary immunity on estimates of vaccine effectiveness using stratified tables. Overall we show that the presence of unmeasured confounder that is related to temporary immunity could possible bias the risk estimates away from the null and that this would result negatively for seasonal influenza (positive VE) and positively for pH1N1 (negative VE). The observed odds ratios are lower than what was observed in the studies by Skowronski *et al*. [1]; however, the direction of effect is consistent such that a protective effect for seasonal influenza and an increased risk for pandemic influenza may be observed. We have to acknowledge that this is an artificial, simplified and constructed scenario with a range of assumptions that are subject to uncertainty. Furthermore, it is possible that the baseline assumptions may differ from what was observed during the pandemic, but we believe them to be within a reasonable range of what is observed through a number of different data sources. This should be conceptualized as a thought experiment that illustrates the effect of confounding that is related to temporary immunity in a way that might be helpful for epidemiologists in the field.

Some unique circumstances during the 2009 pandemic in Canada may have affected the observations made, including the close timing of the first wave in relation to the end of the previous winter season of respiratory infections. Modelling has shown that this timing is critical [16]. It is also worth noting that winter regularly brings a mixture of different influenza and other respiratory viruses. If it is true that the activation of temporary immunity by one respiratory virus might affect susceptibility to others, then this effect may operate to a greater or lesser extent during every influenza season, and would also be likely to vary during any given season. Given the relationship between respiratory infections and vaccination, there is significant potential for this to be an important contributor to unexplained variation in observed vaccine effectiveness. Such variation includes apparent lack of concordance between influenza strain match and vaccine effectiveness. No conclusions can be drawn about the biology of temporary immunity from this study, but these findings support further immunological research into potential mechanisms.

If unmeasured confounding distorts observations of vaccine effectiveness this creates a huge challenge for influenza epidemiologists. The influenza vaccine can be confounded more so than other vaccine exposures due to the fact that the uptake is not universal in all populations. Influenza vaccine uptake will vary according to health care access, indications, occupations and underlying medical conditions, all of which can also influence the likelihood of acquiring influenza.

Confounding is most effectively addressed either through design or through measurement so that we can adjust for its effects during the analysis. It is much harder to design out confounding in observational studies compared to randomized controlled trials. For observational studies on vaccine effectiveness the best solution is to measure confounders and adjust for them in the analysis. This requires greater sample size as well as the ability to accurately measure confounders using feasible and reproducible methods. Other methods to address unmeasured confounding include sensitivity analyses [4,11] and simulation [12,17], which require complex approaches as well as good baseline data into assumptions of potential confounders that exist in the population.

This analysis is subject to baseline assumptions and furthermore did not consider joint or multiplicative effects; thus, may not reflect the situation during the 2009 pandemic. Nevertheless it demonstrates that failure to control for confounders that may bias the associations with the seasonal influenza vaccine (or the pandemic influenza vaccine) will affect the measures of associations. Furthermore these measures of associations can influence different respiratory outcomes (i.e. seasonal influenza versus pandemic influenza) differently.

## Conclusions

Further research is needed into methods that can more completely measure social and behavioural confounders in influenza vaccine effectiveness studies and can be integrated easily into public health surveillance. Until this can be accomplished, questions will always remain about the validity of influenza vaccine effectiveness estimates derived from observational studies.

## Competing interest

The authors declare that they have no competing interests.

## Authors’ contributions

NSC carried out the initial analysis and wrote and edited the manuscript; LCR verified and carried out additional analysis, writing and editing of the manuscript. All authors read and approved the final manuscript.

## Pre-publication history

The pre-publication history for this paper can be accessed here:

http://www.biomedcentral.com/1471-2458/12/458/prepub
